# Identification and functional analysis of LncRNA-XIST ceRNA network in prostate cancer

**DOI:** 10.1186/s12885-022-10007-6

**Published:** 2022-08-29

**Authors:** Jie Wang, Jie Huang, Yingxue Guo, Yuli Fu, Yifang Cao, Kang Zhou, Jianxiong Ma, Bodong Lv, Wenjie Huang

**Affiliations:** 1 The Second Clinical Medical College, Zhejiang Chinese Medical University, Hangzhou, China; 2 College of Pharmaceutical Sciences, Zhejiang Chinese Medical University, Hangzhou, China; 3 Urology Department, Jiaxing First Hospital, Affiliated Hospital of Jiaxing University, Jiaxing, Zhejiang China; 4 Department of Urology, School of Medicine, The Second Affiliated Hospital, Zhejia-Ng University, Hangzhou, China

**Keywords:** Prostate cancer, LncRNA-XIST, ceRNA network, Proliferation, Migration

## Abstract

**Background:**

Long non-coding RNAs (lncRNAs) play a functional role in the progression of prostate cancer (PCa). However, the molecular mechanism, expression, or function of the lncRNA XIST in PCa is not well understood. Therefore, the major goal of this study was to investigate the involvement of XIST in PCa.

**Methods:**

We used the The Cancer Genome Atlas (TCGA) database to conduct a pan-cancer bioinformatics analysis of XIST and identified that it may play an important role in prostate cancer. This finding was verified using clinical samples and in vitro assays. Finally, we constructed an XIST ceRNA network for prostate cancer.

**Results:**

Our in vitro and in vivo results showed that the XIST gene expression level was higher in PCa derived cells and tissues compared to that in normal cells and tissues. XIST gene expression level was positively correlated with the invasion and proliferation of tumour cells. Furthermore, the downregulation of XIST inhibited the growth of subcutaneous 22Rv1 xenografts in nude mice. In addition, we constructed a XIST ceRNA network. Consistent with previous studies, we found that the role of XIST is mediated through via sponges, such as miRNA -96-5p, miRNA -153-3p, and miRNA-182-5p.

**Conclusion:**

High expression level of XIST can lead to enhanced carcinogenicity in PCa. Therefore, XIST has the potential to be used as a prognostic marker and may become a new research focus for the treatment of PCa.

**Supplementary Information:**

The online version contains supplementary material available at 10.1186/s12885-022-10007-6.

## Background

Prostate cancer (PCa), one of the most common malignancies of the male urinary system, is the sixth leading cause of tumour-related deaths in men [1]. PCa is typically asymptomatic in its earliest stage and more common in men over the age of 50, which increases the financial burden on society and has a negative impact on patients' quality of life [1–3]. PCa is characteristically asymptomatic in earliest stage [4], common clinical methods for PCa screening include the detection of prostate-specific antigen levels in blood samples, digital rectal examination, and pelvic magnetic resonance imaging; however, these methods have low diagnostic accuracy [5]. Cytokines and related genes play a role in PCa progression [6, 7], but the molecular mechanisms underlying the development of PCa remain unknown. Exploring the pathogenesis of PCa is therefore critical for developing new diagnostic and therapeutic approaches.

Long noncoding RNAs (lncRNA) are RNAs longer than 200 nucleotides that are not translated [8, 9]. Many biological processes, such as cell growth, migration, invasion, and survival, are affected by lncRNAs [10]. LncRNAs regulate gene expression in eukaryotic cells [11]. Furthermore, there is a close relationship between the abnormal expression of lncRNAs and cancer, suggesting that they have a significant impact on cancer progression and outcome in patients [12]. Changes in lncRNA expression levels have recently been linked to the development of PCa [13]. In cancers, such as colon cancer, pancreatic cancer, and cervical cancer, the levels of the XIST can be elevated and are associated with cell proliferation, apoptosis, and metastasis [14–16]. However, the potential role of XIST in PCa progression and the underlying mechanisms are unknown.

MicroRNAs (miRNAs) are non-coding RNAs consisting of 20 to 30 nucleotides. Further, miRNAs can be competitively sponged by mRNA and lncRNA to form competitive endogenous RNA (ceRNA) networks [17], which can play a tumour-suppressor or tumour-promoting role and are important in the progression of PCa. XIST primarily acts as an miRNA sponge during tumorigenesis [18]. XIST can inhibit the proliferative ability of thyroid cancer cells by targeting the AKT signalling pathway by sponging miRNA-34a [19].

The role of XIST in the progression of PCa is still unclear. Therefore, in this study, we performed a single-gene bioinformatics analysis and built a ceRNA network to investigate the specific role of XIST in PCa.

## Materials and methods

### PCa specimen collection

This experiment followed the Helsinki Declaration and was approved by the Ethics Committee of the Second Affiliated Hospital of Zhejiang University. For the PCa study, 30 PCa tissues and surrounding healthy prostate tissues were obtained from the hospital. All specimens were excised, washed with sterile normal saline, and stored in liquid nitrogen for subsequent use. All PCa patients completed informed consent forms and had not undergone radiation or chemotherapy prior to surgery.

### Quantitative real-time polymerase chain reaction (qRT-PCR)

The total RNA was extracted from PCa tissues and cells using the TRIzol (Thermo Fisher Scientific, Waltham, MA, USA) method according to manufacturer's instructions. Total RNA was reverse transcribed by PrimeScript™ RT Master Mix (Takara, Beijing, China). qPCR process was performed on the resultant cDNA using Applied Biosystems 7500 real-time PCR system (Foster City, CA, USA) and TB Green Premix Ex Taq ™ II (Takara). The PCR primer sequences were as follows: XIST: forward: 5ˊ-TGGACTCAGTAACACCCCTTT-3ˊ, reverse: 5ˊ-GGACAATGACG AAGCCACTTA-3ˊ. Actin, forward: 5ˊ-AGCGAGCATCCCCCAAAGTT-3ˊ, reverse: 5ˊ-GGGCACGAAGGCTCATCATT-3ˊ. XIST levels were normalised to actin levels using the 2^−ΔΔCt^ method.

### Bioinformatics analysis of XIST

To further understand the role of XIST in tumours, we conducted a pan-cancer study. A standardised universal cancer dataset was obtained from the University of California Santa Cruz Xena database (https://xenabrowser.net/) and data from The Cancer Genome Atlas (TCGA) pan-cancer (PANCAN, *N* = 10,535, G = 60,499) database enabled us to obtain the expression levels of XIST (ENSG00000229807) for every sample. The expression level differences between normal and tumour samples in each patient were calculated using R software (Version 3. 6. 4, limma package) [20] and the significance was determined using unpaired Wilcoxon rank-sum and signed-rank tests. The CD4^+^ T cell, CD8^+^ T cell, B cell, macrophage, neutrophil, and dendritic cell infiltration scores of prostate cancer patients were revaluated using R software (IOBR, and psych package) [21, 22] and the Timer method. Additionally, we obtained the DNAss tumour dry score based on the methylation characteristics of each tumour. Finally, we used the R maxstat package [23], to calculate the optimal truncation value of XIST. We set the minimum sample number to be greater than 25% and the maximum sample number to be less than 75% and obtained the optimal truncation value as of-3. 406,112. Based on this truncation value, patients were divided into high-expression and low-expression groups according to the expression level of XIST. The Survfit function of the R software package Survival [24] was used to evaluate the prognosis of patients based on XIST gene expression and log-rank test was used for significance.

### Cell culture

The normal human prostate epithelial cell line RWPE1 and the human PCa cell lines VCaP and 22Rv1 were purchased from the Center of Molecular Excellence of Chinese Academy of Sciences (Shanghai, China). Cells were cultured in a complete medium consisting of Dulbecco’s modified Eagle’s medium (DMEM; Thermo Fisher Scientific, Waltham, MA, USA) containing 10% foetal bovine serum (FBS, Thermo Fisher Scientific), 1% penicillin–streptomycin solution (Biosharp, Hefei, China), and incubated in 5% CO_2_ at 37 °C.

### Lentivirus production and cell transfection

GenePharma(Shanghai, China) designed and synthesised adenoviral short hairpin XIST (XIST-shRNA1 and XIST-shRNA2) plasmids. The targeting shRNA sequences were: XIST-shRNA1, 5ˊ- -3ˊ; XIST-shRNA2, 5ˊ- -3ˊ. Adenoviral XIST was inserted into pcDNA3.1 to produce an XIST overexpression vector (Ad-XIST, GenePharma). An empty pcDNA3.1 vector was used as a negative control (NC). The plasmids XIST-shRNA1, XIST-shRNA2, and Ad-XIST were transfected into 293 T cells. The supernatant containing the retroviral particles was collected 72 h after transfection. VCaP cells were then incubated for 3 days with NC or Ad-XIST supernatant. Meanwhile, 22Rv1 cells were transfected for 3 days with NC, XIST-shRNA1, and XIST-shRNA2 supernatants. After three days incubation, the cells were harvested, total RNA extracted and the concentration of XIST.1was determined using qRT-PCR.

### Cell Counting Kit-8 (CCK-8) assay

The viability of the cells was determined using the CCK-8 (Beyotime Institute of Biotechnology, Shanghai, China) according to the manufacturer’s instructions. An average of 5,000 VCaP or 22Rv1 cells were incubated overnight at 37 ℃. The cells were then transfected with Ad-XIST or XIST-siRNA1 and incubated for 1 day, 2 days and 3 days. CCK-8 reagent (10 μL) was then added to each well and incubated for the appropriate time (1–4 h). The absorbance was measured at 450 nm using a microplate reader (Bio-Tek Instruments Inc., Winooski, VT, USA).

### Immunofluorescence staining assay

PCa cells were transfected with Ad-XIST or XIST-siRNA1 and incubated for three days in 5% CO_2_ incubator at 37 °C. The cells were washed three times with phosphate buffered saline (PBS), then fixed with 4% paraformaldehyde (Macklin, Shanghai, China) for 30 min, washed with PBS three times, and treated with 0.2% Triton X-100 (Thermo Fisher Scientific) for 10 min. The cells were mixed with the primary antibody against Ki 67 (1: 100, Abcam Cambridge, MA, USA) and incubated at 4 ℃ overnight. After washing in TBST (applygen, Beijing, China), the cells are then incubated with an anti-rabbit second antibody (1:1000; Abcam). Nuclear staining with 4ˊ,5-diamidino-2-phenylindole (Thermo Fisher Scientific) was performed for 5 min. The fluorescence signal was observed using a laser confocal microscope (Olympus CX23, Tokyo, Japan).

### Transwell invasion assay

PCa cells were diluted to 2 × 10^4^/mL in order to conduct the cell invasion test. PCa cells (200 μL inDMEM) were seeded into the upper chamber of a 24 well Transwell insert (Corning, NY, USA) coated with matrix gel (BD Biosciences, Franklin Lake, NJ, USA). To induce cell invasion, 600 μL of complete DMEM containing 10% FBS was added to the lower chamber. After 24 h, cotton swabs were used to remove the cells adhering to the upper surface of the compartment The cells on the lower surface of the insert were the stained with 0.2% crystal violet (Macklin). After washing with PBS, the cells were examined by laser confocal microscope.

### Flow cytometry

The apoptosis state of VCaP cells after the silencing of XIST was detected by flow cytometry. The cell supernatants were centrifuged together with the trypsinised cell suspension and stained with Annexin V-FITC (Thermo Fisher Scientific). Finally, the BD FACS Calibur System™ (BD Biosciences) was used for flow cytometry detection.

### Western blotting

Radioimmunoprecipitation assay buffer (Abcam) and 1% protease inhibitor (Solarbio, Beijing, China) were used to extract total protein from VCaP cells. Proteins were separated using 12% sodium dodecyl sulphate–polyacrylamide gel electrophoresis before transferring to polyvinylidene fluoride (PVDF; Millipore, Burlington, MA, USA) membrane using theconstant current wet transfer method of 350 mA for 60 min. Membranes were then blocked with 5% skimmed milk (BD Biosciences) for 2 h at 20–27 °C. After the blocking was complete, the PVDF membrane was cut according to the molecular weight of protein, and incubated with the corresponding primary antibodies anti-Bax (1: 1000, Abcam), anti-Bcl-2 (1:500, Abcam), anti-active caspase 3 (1:1000, Cell Signaling Technology, Danvers, MA, USA), or anti-β-actin (1:2000, Cell Signaling Technology) overnight at a 4 °C. After washing in TBST, the membranes were then incubated for 2 h at20–27 °C with a horseradish peroxidase-conjugated goat anti-rabbit and anti-mouse IgG secondary antibody (1:10,000, Abcam). The proteins were detected using the enhanced chemiluminescence (Biomiga, San Diego, California, USA). Finally, ImageJ software (1.48v) [25] was used for semi-quantitative analysis. Each experimental operation was verified three times, with β-actin as the reference gene.

### In vivo* tumorigenesis*

Four-week-old (12–14 g) male BALB/c nude mice (*n* = 9) were purchased from Shanghai, SLAC (Shanghai, China) and reared in a barrier environment at the Animal Experimental Center of Zhejiang University of Chinese Medicine. The animals were randomly divided into three groups: blank, NC, and XIST-shRNA1. XIST-shRNA1-transfected 22Rv1 cells (5 × 10^6^ cells per mouse) were injected into the left axilla of the XIST-shRNA1 nude mice group, at the same time, the blank group was inoculated with the 22Rv1 cells (5 × 10^6^ cells per mouse) transfected with the blank vector, and the normal group was injected with the untreated 22Rv1 cell line. Every week, the tumour burden was calculated V = (length × width^2^)/2. After four weeks, mice in each group were euthanised under anaesthesia and all tumours were removed. The tumour weights were then measured. The number of replicating cells were determined by immunostaining with a Ki67 antibody. For each section, Ki67 positivity was measured in three high-power fields. Apoptotic cells were identified using a TUNEL kit (Abcam Cambridge, MA, USA), using manufacturer’s instructions and analysed in 4–6 random fields.

### Construction of ceRNA network for XIST

Weighted correlation network analysis (WGCNA package) [26] and differential expression analysis (limma package) were used to analyse the miRNAs in prostate cancer and para-cancerous tissues in TCGA to further clarify the possible role of XIST in PCa. The miRNAs obtained intersected with those predicted by lnCAR, resulting in the identification of the desired miRNAs. The target mRNAs for miRNA-93-5p, miRNA -96-5p, miRNA -153-3p, miRNA-182-5p, miRNA-200c-3p and miRNA-615-3p were predicted using starBase [27] and miRDB [28]. To identify potential ceRNAs, the predicted target mRNAs were intersected with the differentially expressed mRNAs obtained from limma analysis of prostate cancer and adjacent tissues from TCGA. Additionally, Lnc2Cancer 3. 0 [29] was used to predict transcription factors that may regulate XIST. Finally, the entire ceRNA network was visualised using Cytoscape (Version 3.7.1) [30].

### Statistical analysis

Three biological replicates were independently performed for all experiments, and all statistics were performed in SPSS Statistics for Windows, version 16.0 (SPSS Inc., Chicago, IL, USA). T-test or one-way analysis of variance was used for comparison between groups (*p* < 0.05). The data were visualized using GraphPad Prism software 8.0(GraphPad Software, San Diego, CA, USA).

## Results

### XIST pan-cancer analysis

**Fig. 1:**
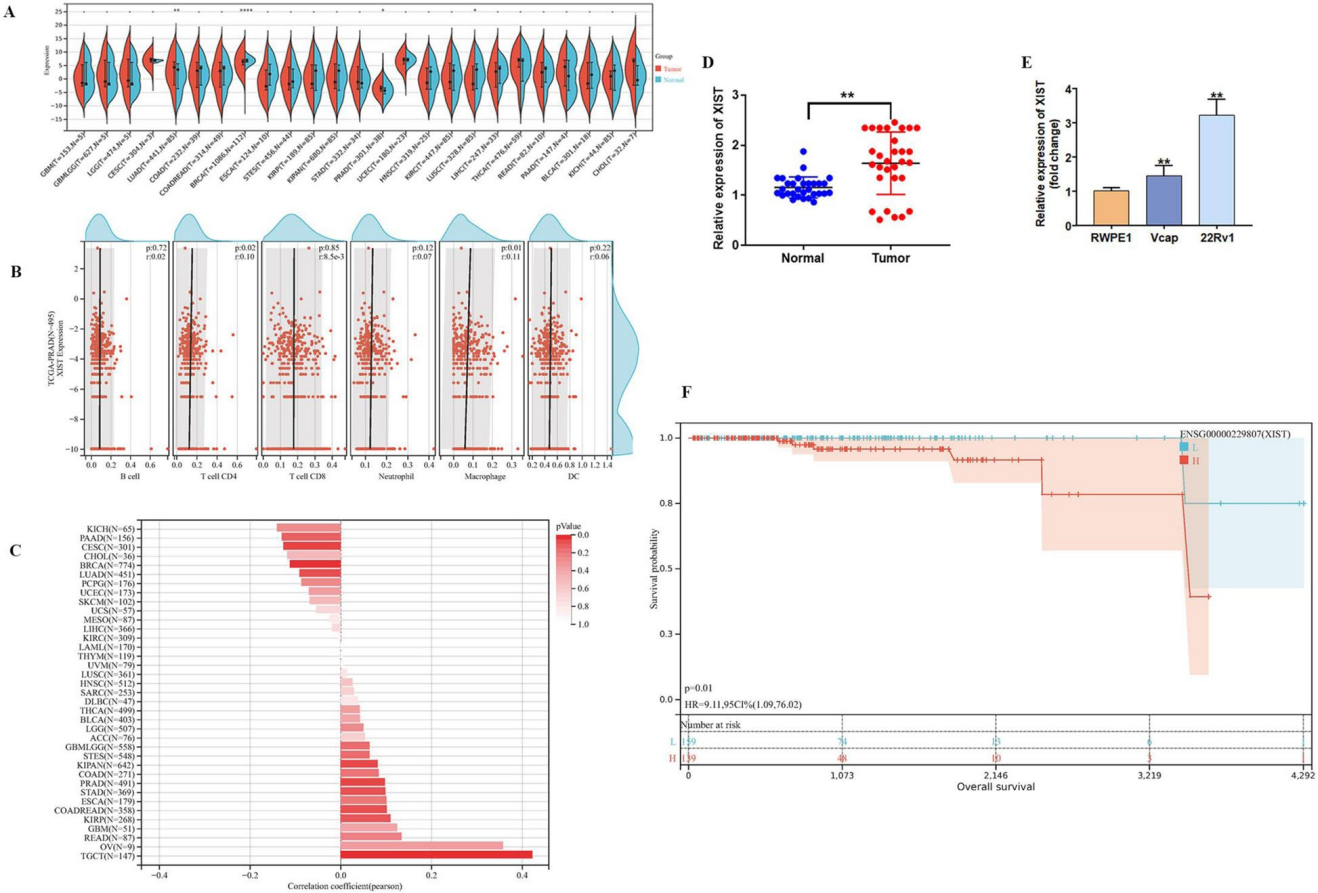
Expression of lncRNA XIST in tumour A. The differential expression of lncRNA XIST in various tumours in TCGA database. Red represents the expression in tumours, and blue represents the expression in paracancerous tissues. B. LncRNA XIST gene expression was positively correlated with CD8 + T-cell and macrophage expression in the TCGA database. C. There was a positive correlation between lncRNA XIST gene expression and tumour cell stemness in prostate cancer in TCGA database. D. The expression levels of lncRNA XIST in 30 PCa tissues and surrounding healthy prostate tissues were detected using qRT‒PCR. E. The level of lncRNA XIST in the human normal prostate epithelial cell line RWPE1 and human PCa cell lines Vcap and 22Rv1 was detected using qRT‒PCR. F. High expression of lncRNA XIST in prostate cancer patients indicates poor prognosis

We performed a pan-cancer analysis of XIST and discovered that significant upregulation was observed in two types of tumours, LUAD (Tumour:2. 56 ± 4. 63, Normal:1. 18 ± 4. 86, *P* = 4. 1 × 10–3) and PRAD (Tumour:-3. 64 ± 1. 55, Normal:-4. 19 ± 1. 94, *P* = 0. 01). BRCA (Tumour:6. 08 ± 1. 64, Normal:6. 81 ± 0. 85, *P* = 6. 7 × 10–6) and LUSC (Tumour:-0. 10 ± 4. 75, Normal:1. 18 ± 4. 86, *P* = 0. 04) were found to be significantly downregulated (Fig. 1A). Immune infiltration was significantly correlated with TCGA\BLCA (Bladder Urothelial Carcinoma), TCGA\BRCA (Breast invasive carcinoma), TCGA\HNSC (Head and Neck squamous cell carcinoma), TCGA\KIPAN (Pan-kidney cohort (KICH + KIRC + KIRP)), TCGA\KIRC (Kidney renal clear cell carcinoma), TCGA\KIRP (Kidney renal papillary cell carcinoma), TCGA\LIHC (Liver hepatocellular carcinoma), TCGA\LUAD (Lung adenocarcinoma), TCGA\LUSC (Lung squamous cell carcinoma), TCGA\MESO (Mesothelioma), TCGA\OV (Ovarian serous cystadenocarcinoma), TCGA\PCPG (Pheochromocytoma and Paraganglioma), TCGA\PRAD (Prostate adenocarcinoma), TCGA\READ (Colon adenocarcinoma/Rectum adenocarcinoma Esophageal carcinoma), TCGA\SARC (Sarcoma), TCGA\STAD (Stomach adenocarcinoma), TCGA\STES (Stomach and Esophageal carcinoma), TCGA\TGCT (Testicular Germ Cell Tumors), and TCGA\UCEC (Uterine Corpus Endometrial Carcinoma) (Fig. 1B, Table 1). The analysis of cancer stemness revealed that XIST was significantly correlated across five tumour types, with a significant positive correlation in KIPAN, PRAD, and TGCT and a significant negative correlation in CESC and BRCA (Fig. 1C). XIST also showed high expression levels in prostate cancer patients (Fig. 1D) which was also consistent with of the results from the prostate cancer cell lines (Fig. 1E). Finally, we discovered that XIST expression levels differed significantly in patients with different prostate cancer prognoses; the higher the expression level of XIST, the poorer the prognosis of the patients. (Fig. 1F).

**Table 1:** TCGA Study abbreviations

Study Abbreviation	Study Name
BLCA	Bladder Urothelial Carcinoma
BRCA	Breast invasive carcinoma
HNSC	Head and Neck squamous cell carcinoma
KIPAN	Pan-kidney cohort (KICH + KIRC + KIRP)
KIRC	Kidney renal clear cell carcinoma
KIRP	Kidney renal papillary cell carcinoma
LIHC	Liver hepatocellular carcinoma
LUAD	Lung adenocarcinoma
LUSC	Lung squamous cell carcinoma
MESO	Mesothelioma
OV	Ovarian serous cystadenocarcinoma
PCPG	Pheochromocytoma and Paraganglioma
PRAD	Prostate adenocarcinoma
READ	Colon adenocarcinoma/Rectum adenocarcinoma Esophageal carcinoma
SARC	Sarcoma
STAD	Stomach adenocarcinoma
STES	Stomach and Esophageal carcinoma
TGCT	Testicular Germ Cell Tumors
UCEC	Uterine Corpus Endometrial Carcinoma

### Overexpression of XIST promotes proliferation and invasion of PCa cells

**Fig. 2:**
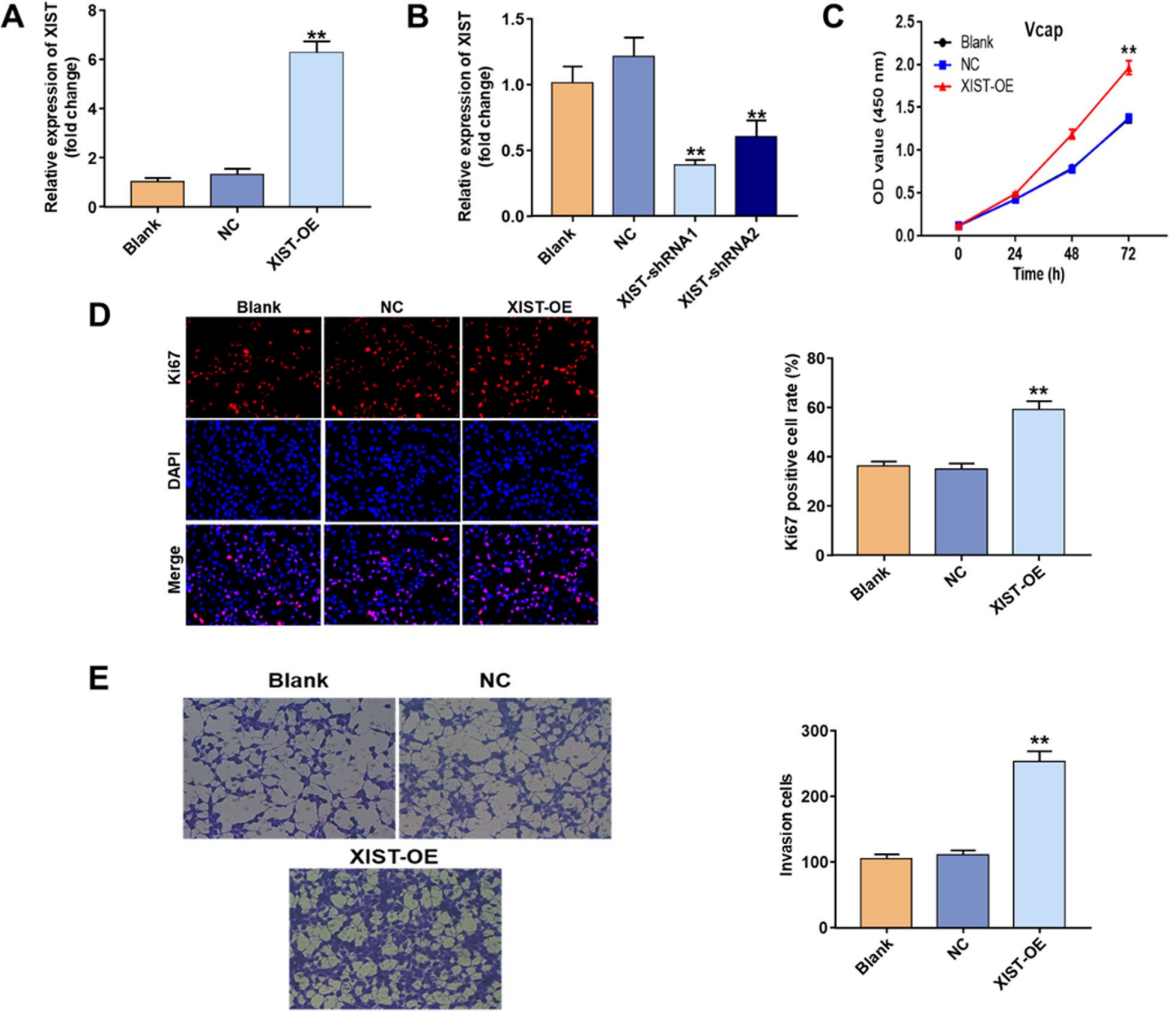
LncRNA XIST was upregulated in PCa tissues, and cell lines can promote the proliferation and invasion of PCa cells. **A** VCaP cells were transfected with NC or Ad-XIST for 72 h. The expression of lncRNA XIST in transfected VCaP cells was detected by qRT-PCR. **B** 22Rv1 cells were transfected with NC, XIST-shRNA1 or XIST-shRNA2 for 72 h. The level of XIST in 22Rv1 cells was detected by qRT‒PCR. **P<0.01 vs NC group. **C** VCaP cells were transfected with NC or Ad-XIST for 24, 48 and 72 h. The cell viability in transfected VCaP cells was detected by CCK8 assay. **D** VCaP cells were transfected with NC or Ad-XIST for 72 h. Relative fluorescence expression levels were quantified by Ki67 and DAPI staining. **E** VCaP cells were transfected with NC or Ad-XIST for 24 h. The cell migration in transfected VCaP cells was detected by transwell assay. **P<0.01 vs. NC group

The XIST gene was highly expressed in 22Rv1 cells but poorly expressed in VcaP cells. Because of their low levels of XIST expression, we stably overexpressed XIST in VcaP cells (Fig. 2A). We used two siRNAs (XIST-siRNA1 and XIST-siRNA2; the level of XIST was markedly reduced in 22Rv1 cells after transfection with XIST-shRNA1, as shown in Fig. 2 B. As a result, XIST-siRNA1 was used in subsequent experiments. The results of CCK-8 and immunofluorescence showed that the proliferation and invasiveness of cells were positively correlated with the expression level of XIST (Fig. 2C–E).

### Downregulation of XIST inhibited invasion and proliferation but induced apoptosis of 22Rv1 cells

**Fig. 3:**
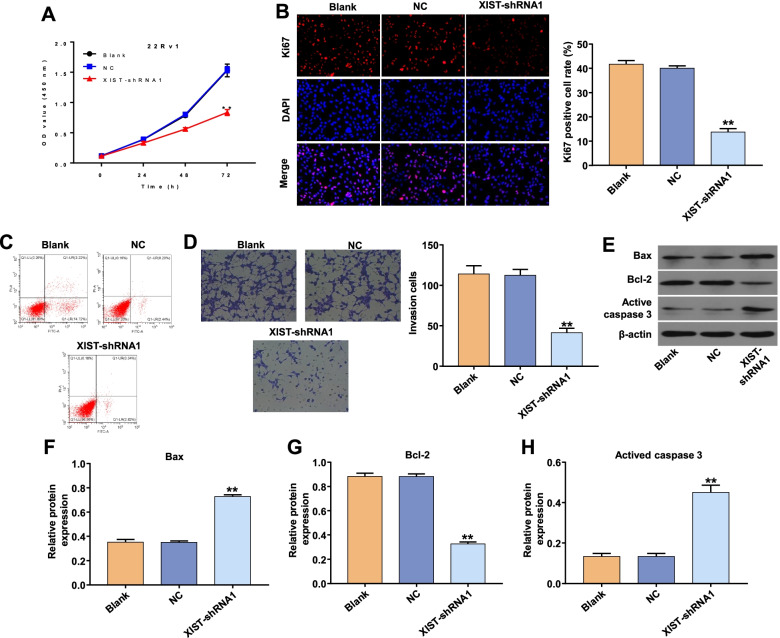
Downregulation of lncRNA XIST inhibited proliferation and invasion but induced apoptosis of PCa cells. (A) Vcap cells were transfected with NC or XIST-shRNA1 for 24, 48 and 72 h. A CCK-8 assay was used to measure cell viability. (B) Vcap cells were transfected with NC or XIST-shRNA1 for 72 h. Relative fluorescence expression levels were quantified by Ki67 and DAPI staining. (C) Vcap cells were transfected with NC or XIST-shRNA1 for 24 h. Cell invasion was detected using a transwell invasion assay. (D) Vcap cells were transfected with NC or XIST-shRNA1 for 72 h. Apoptotic cells were detected with Annexin V and PI double staining. (E) The expression levels of Bax, Bcl-2, and active caspase 3 in 22Rv1 cells were detected with western blotting. β-actin was used as an internal control. (F, G, H) The relative expression levels of Bax, Bcl-2, and active caspase 3 in 22Rv1 cells were quantified via normalisation to β-actin. **P < 0.01 vs. NC group

The downregulation of XIST in 22Rv1 cells by transfection with XIST-shRNA1 caused significant inhibition of proliferation compared with the NC group (Fig. 3A and 3B), XIST silencing markedly reduced the invasion ability of 22Rv1 cellscompared with the NC group (Fig. 3C). Furthermore, when 22Rv1 cells were transfected with XIST-shRNA1 the apoptotic rate was notably higher than in the NC group (Fig. 3D). XIST inhibition significantly upregulated apoptosis-related proteins such as Bax, active Caspase 3 and anti-apoptosis factor Bcl2 (Fig. 3E–H). These findings indicate that XIST downregulation inhibits invasion and proliferation but increases apoptosis in PCa cells.

### Downregulation of XIST inhibited the tumorigenesis of 22Rv1 right axillary transplanted tumour in vivo

**Fig. 4.**
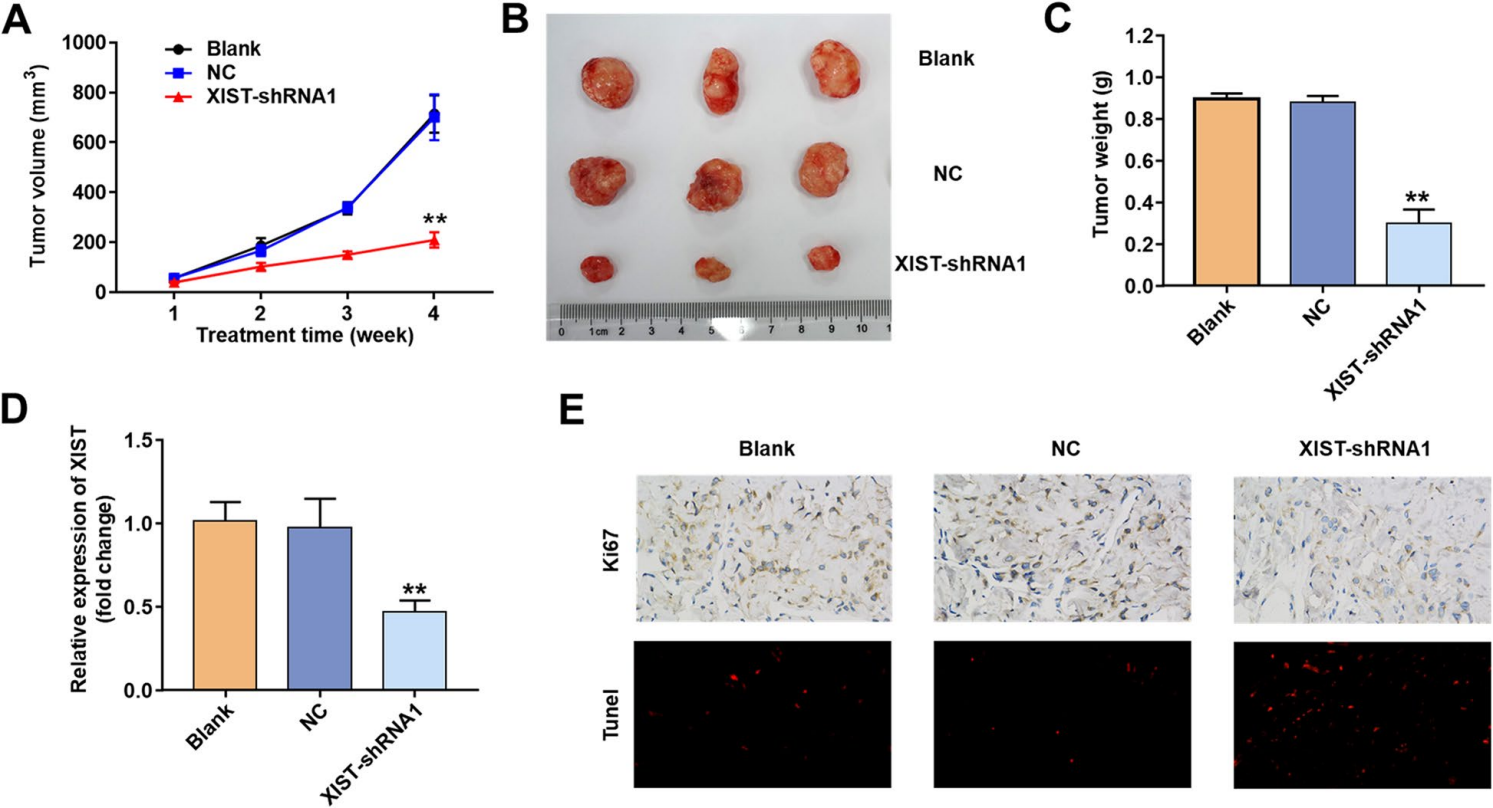
22Rv1 cells were subcutaneously implanted with XIST-shRNA1-infected cells to establish a xenograft model of PCa. (A) Tumour volumes of animals were measured weekly. (B) Representative photographs of xenograft tumors were taken after 4 weeks. (C) Tumour weights in each animal were calculated. (D) qRT‒PCR was used to detect the level of lncRNA XIST in tumour tissues. (E) Knockdown of lncRNA XIST significantly decreased the percentage of Ki-67-positive cells and increased the number of apoptotic cells in the tumours compared with the NC group (200 ×) **P < 0.01 vs. NC group

To investigate the role of XIST in vivo, XIST-shRNA1-transfected cells were injected subcutaneously into the right axilla of nude mice (10 × 10^5^ cells/mice). Downregulation of XISTsignificantly reduced the tumour burden compared to the NC group (Fig. 4A and 4B). Furthermore, the tumour weight in the XIST-shRNA1 group was significantly lower (Fig. 4C). After transfection with XIST-shRNA1, the level of XIST expression in tumour tissues was significantly reduced (Fig. 4D). Figure 4E shows that the XIST-shRNA1 group had significantly lower tumour proliferation and higher apoptosis than the NC group. Our results show that down-regulation of XIST decreases tumorigenesis of 22Rv1 subcutaneous xenografts in vivo.

### Construction of ceRNA networks

**Fig. 5:**
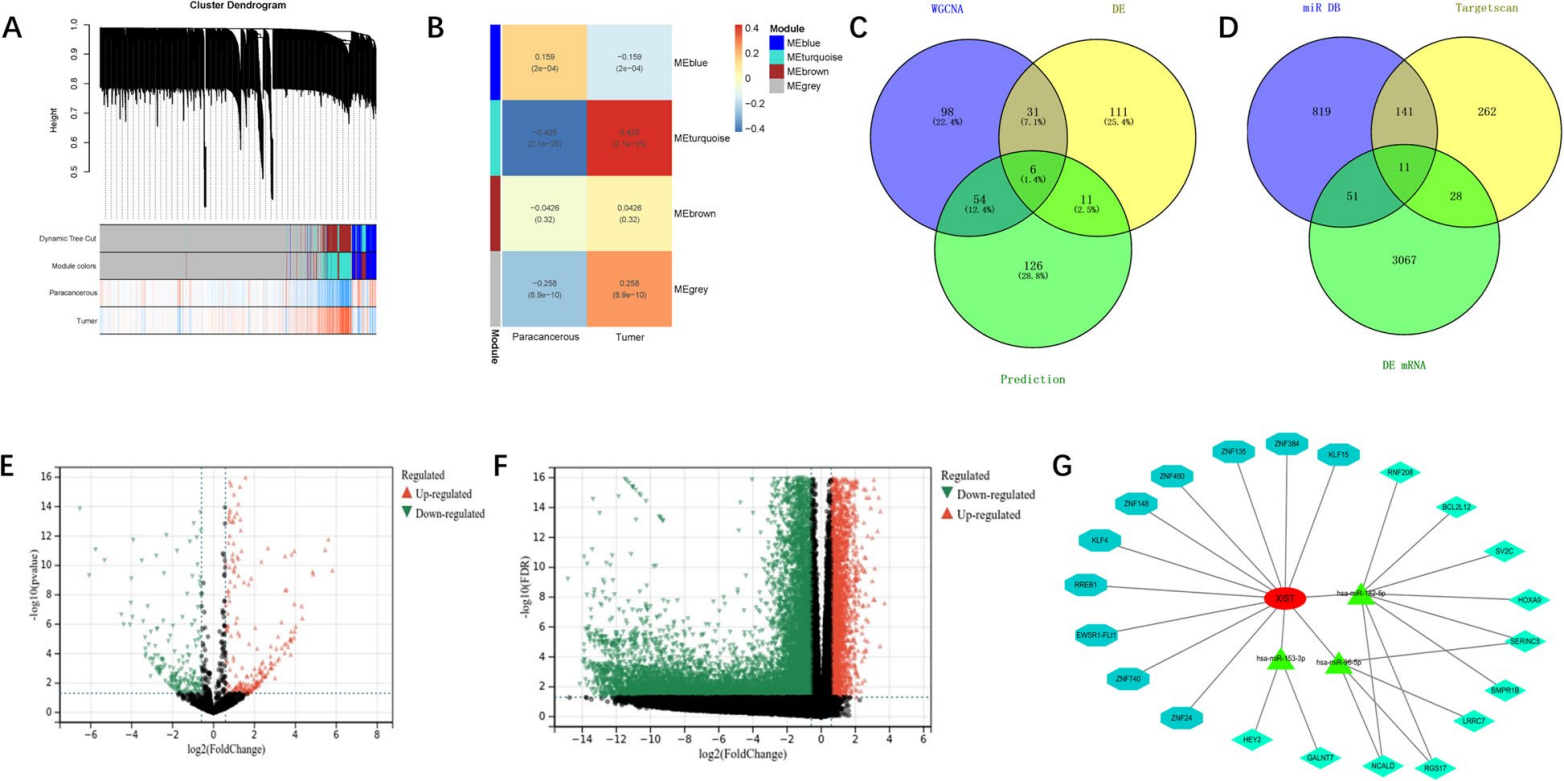
TCGA database data analysis results and ceRNA network. (A-B) WGCNA of miRNAs in prostate cancer and adjacent tissues in the TCGA database. (C) WGCNA showed that miRNAs, differentially expressed miRNAs, and predicted miRNAs intersected. (D) Intersection of differentially expressed mRNAs and predicted mNRAs. (E) Volcano plot of differentially expressed miRNAs in prostate cancer and adjacent tissues in the TCGA database. Green represents downregulation, and red represents upregulation. (F) Volcano plot of differentially expressed mRNAs in prostate cancer and adjacent tissues in the TCGA database. Green represents downregulation, and red represents upregulation. (G) CeRNA network of XIST

The ceRNA network is the most important functional mode of lncRNAs in the pathogenesis of prostate cancer and other tumours [31, 32], therefore the downstream targets of XIST were predicted and the ceRNA network was built. First, we used WGCNA to perform a cluster analysis of miRNA sequencing results in TCGA data (Fig. 5A) and selected the blue module with the highest correlation with tumours for further investigation. The blue module contained a total of 134 miRNAs (Fig. 5B). Subsequently, the expression differential of miRNA between healthy and tumour was calculated using R software (Version 3. 6. 4). In TCGA prostate cancer, 445 differentially expressed miRNAs (236 upregulated and 182 downregulated; logFC > 2 adjP < 0.05) were found (Fig. 5E). WGCNA was merged with differentially downregulated miRNAs and miRNAs predicted by lnCAR to yield six miRNAs (Fig. 5C). Similarly, we obtained the differentially expressed mRNAs from TCGA database, including 3157 upregulated mRNAs and 14,263 downregulated mRNAs (Fig. 5F). The Targetscan, miRDB prediction, and differential expression mRNA results in TCGA database were used to identify the 11 target mRNAs (Fig. 5D). The transcription factors KLF4, RREB1, EWSR1-FLI1, ZNF740, ZNF24, ZNF384, KLF15, ZNF135, ZNF460, and ZNF148, were predicted to regulate XIST by Lnc2Cancer 3. 0. Finally, we established the XIST ceRNA network, which included three miRNAs, eleven mRNAs, and ten transcription factors (Fig. 5G).

## Discussion

Although there are numerous clinical treatments for prostate cancer, including surgical resection, hormone therapy, radiotherapy, and immunotherapy, the disease is highly heterogeneous and has a poor prognosis after treatment [33]. Therefore, it is essential to investigate new pathological mechanisms of PCa to find effective treatments, thereby slowing the incidence rate of cancer, lowering the mortality rate of cancer, and improving prognosis and survival rates.

The roles of lncRNAs in the occurrence and development of various human malignant tumours, including PCa, have been established [34, 35]. Abnormal expression of XIST is commonly found in human malignancies [31, 36, 37]. For example, upregulation of XIST promotes progression by regulating miRNA140-5p and OCR1 [36]. Similarly, XIST regulates ZEB1 expression in pancreatic cancer by sponging miRNA-429 to promote migration, invasion, and EMT [37]. However, XIST can be detected in normal prostate tissues, although its expression level is higher in PCa [38]. We found that in the sequencing data from TCGA database, XIST levels were higher in PCa tissues than in para-cancerous tissues. This may be due to increased levels of demethylation of the XIST 5ˊ region in patients with prostate cancer, resulting in the increased expression level of XIST [39]. Therefore, we speculate that XIST may contribute to an increased carcinogenicity. In addition, survival analysis revealed that the upregulated expression of XIST had a poor prognosis.

Following tumour immune cell infiltration and tumour stem cell analysis, researchers discovered that XIST expression was linked to tumour progression in PCa and was upregulated in clinical PCa tissue samples and cell lines. Our in vitro experiments showed that the expression level of XIST was positively correlated with the invasion and proliferation of prostate cancer cells, but negatively correlated with apoptosis. In vivo experiments showed that inhibiting the expression of XIST could delay the progression of the tumour after subcutaneous inoculation in nude mice. Finally, our experimental results suggest that the XIST is an oncogene that plays a role in the genesis and progression of PCa.

CeRNA networks, which include sponge miRNAs that compete with lncRNAs, have been implicated in the progression of several tumours, including prostate cancer. Lnc01679 slows the progression of PCa by modulating the miRNA-3150a-3p /SLC17A9 axis [40]. PCAT1 stimulates PCa proliferation via c-MYC by sponging miRNA-3667-3p, and FSCN1 stimulates PCa proliferation via miRNA-145-5p [41]. However, research on XIST in the prostate ceRNA network has seldom been reported.

In this study, we constructed an XIST ceRNA network to investigate the potential regulatory mechanism by which XIST influences prostate cancer growth. Consistent with previous studies, we found that it may function via the sponges miRNA -96-5p, miRNA -153-3p, and miRNA-182-5p. XIST regulates breast cancer progression by targeting miRNA-182-5p [42, 43]. We also discovered that miRNA -96-5p plays a role in a variety of tumours [44], including prostate cancer. Further, miRNA -96-5p is expressed at low levels in prostate tumour tissues, and its level of expression is negatively correlated with the Gleason score, which confirms our prediction [45]. In addition, miRNA-153-3p improves radiosensitivity in human glioma cells by targeting BCL2 [46]. Downregulation of XIST in our study may have an anti-tumour effect by regulating miRNA-153-3p/BCL2. In summary, XIST may regulate tumour progression by regulating the expression of miRNA-96-5p, miRNA-153-3p, and miRNA-182-5p; however, its complex regulatory mechanism requires further experimental validation.

## Conclusions

**Fig. 6:**
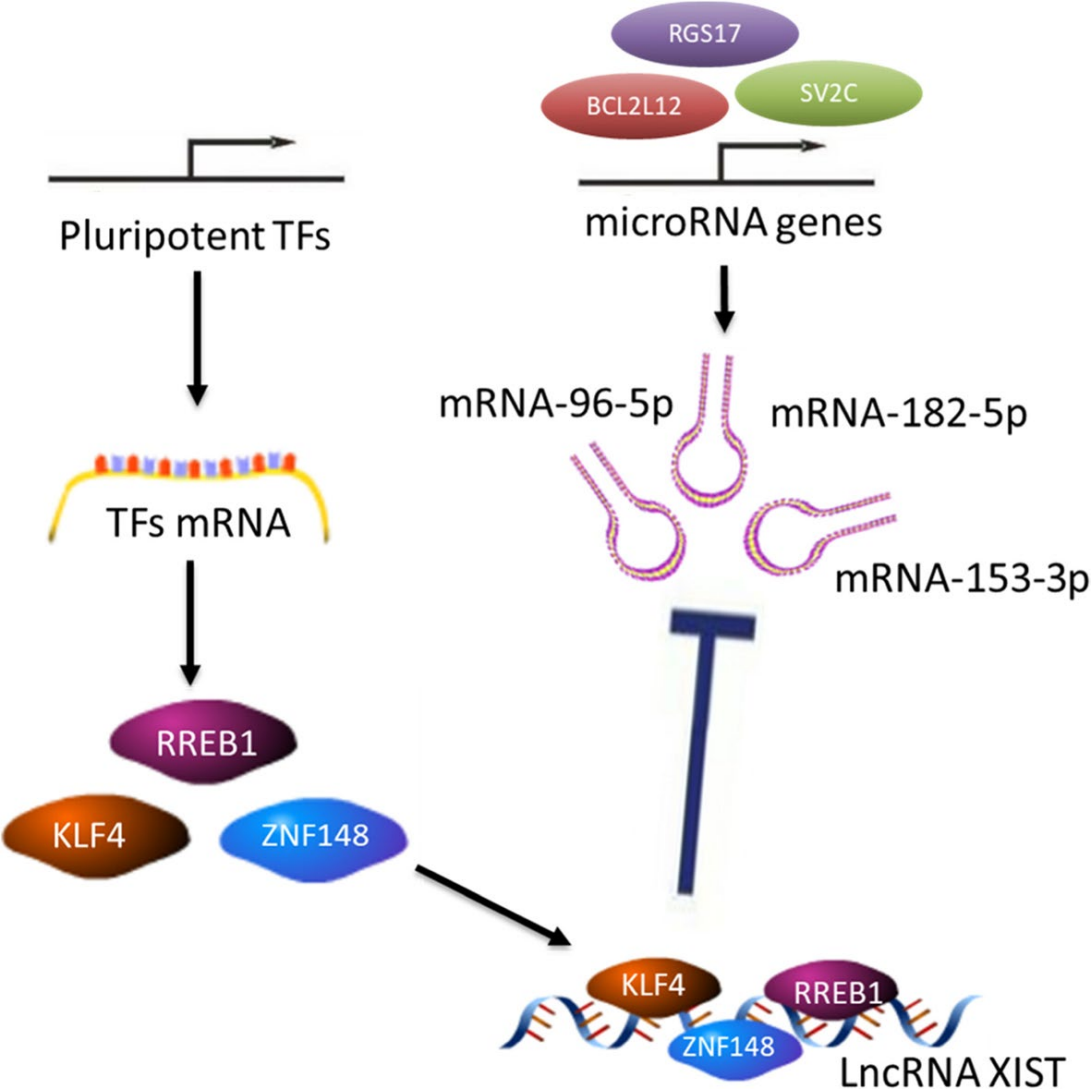
Mechanism hypothesis diagram

Our study found that XIST may not predict whether patients will develop prostate cancer but is highly expressed in PCa and its expression is related to patient prognosis. Through in vivo and in vitro experiments, we found that the overexpression of XIST improved the invasion and proliferation of PCa cell lines, whereas knockdown inhibited tumour cells from acquiring these abilities. Furthermore, we constructed a ceRNA network for XIST using bioinformatics methods to determine its potential regulatory mechanism and discovered that miRNA-96-5p, miRNA-153-3p and miRNA-182-5p may be regulated by XIST in PCa (Fig. 6).

## Supplementary Information

**Additional file 1.** 

**Additional file 2.** 

**Additional file 3.** 

**Additional file 4.** 

**Additional file 5.** 

**Additional file 6.** 

**Additional file 7.** 

**Additional file 8.** 

**Additional file 9.** 

**Additional file 10.** 

## Data Availability

The datasets generated and analysed during the current study are available in TCGA data portal (https://portal.gdc.cancer.gov/projects/TCGA-PRAD). Analysed data is available within supplementary information or from the authors upon reasonable request.
