# Supplementary figures and images for: Identification and functional analysis of LncRNA-XIST ceRNA network in prostate cancer
Source: BMC Cancer. 2022 Aug 29;22:935. doi: 10.1186/s12885-022-10007-6 (PMC9426231; doi:10.1186/s12885-022-10007-6)

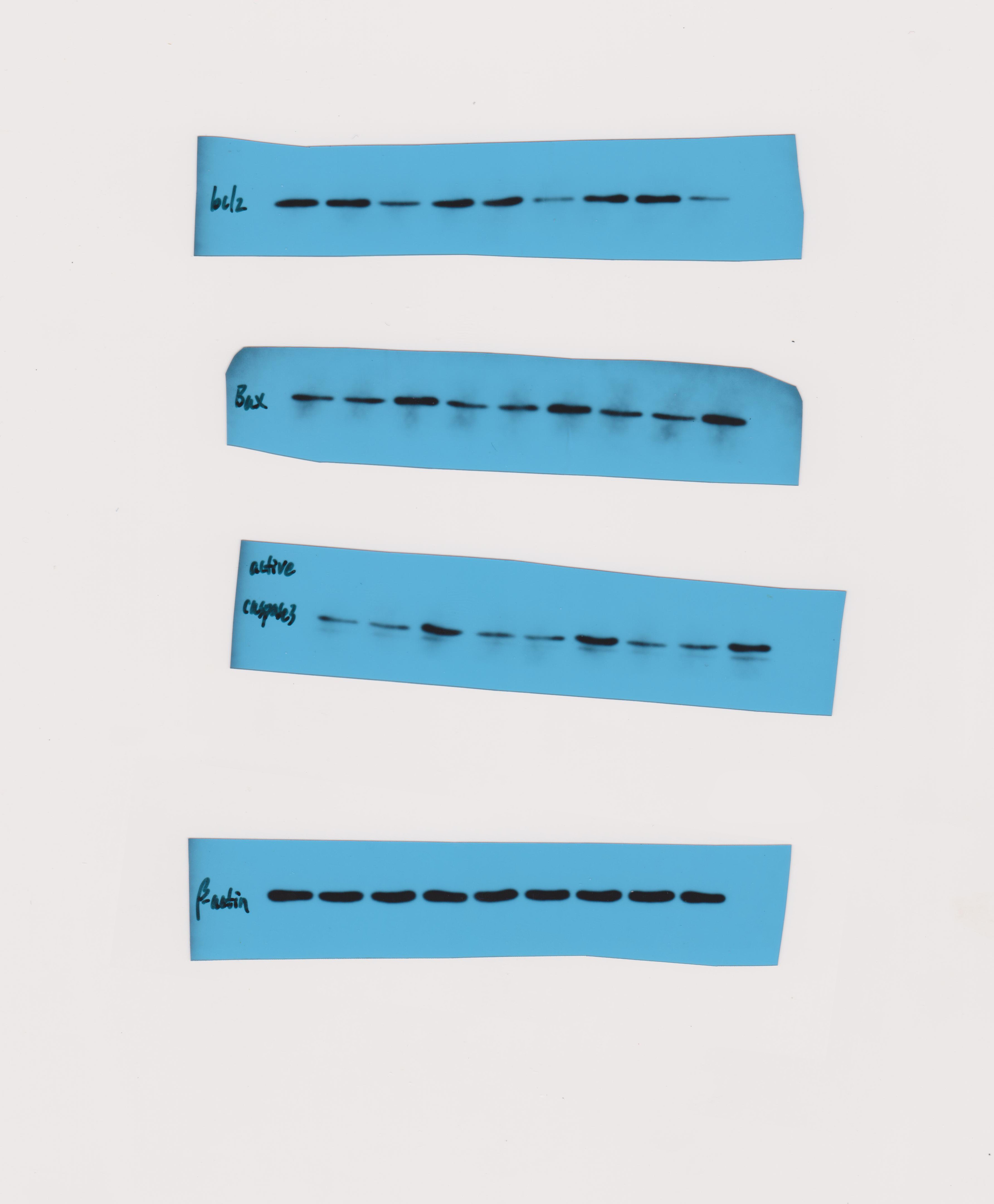

Supplement: Supplementary file 10 — Additional file 10. [file 12885_2022_10007_MOESM10_ESM.jpg]
